# Gut microbiota and metabolic characteristics in PWHIV with metabolic dysfunction-associated steatotic liver disease (MASLD)

**DOI:** 10.1080/21505594.2026.2739091

**Published:** 2026-10-05

**Authors:** Huiting Liu, Tianzhen Xu, Yongzheng Guo, Xiaotang Zhou, Chunhui Yan, Biao Zhu, Yirui Xie

**Affiliations:** a State Key Laboratory for Diagnosis and Treatment of Infectious Diseases, National Clinical Research Center for Infectious Diseases, Collaborative Innovation Center for Diagnosis and Treatment of Infectious Diseases, The Department of Infectious Diseases, The First Affiliated Hospital, School of Medicine, Zhejiang University, Hangzhou, People’s Republic of China; b The Department of Infectious Diseases, The First People’s Hospital of Linping District, Hangzhou, People’s Republic of China

**Keywords:** HIV, metabolic dysfunction-associated fatty liver disease, gut microbiota, metabolomics, immune, oxidative stress

## Abstract

The global prevalence of Metabolic dysfunction-associated steatotic liver disease (MASLD) among persons with HIV (PWHIV) is estimated at 34%. This study examined the influence of gut microbial and metabolic alterations in PWHIV with MASLD. We conducted 16S rRNA sequencing on 60 fecal samples (24 from HIV group and 36 from HIV-MASLD group) to assess microbial composition, and employed LC/MS-based metabolomics on both fecal and plasma samples to identify variations in metabolites. An exploratory analysis was performed to compare plasma cytokines and oxidative stress markers between the two groups. Sequencing in the HIV-MASLD group showed lower *Faecalibacterium* and *Anaerobutyricum*, and higher *Klebsiella*, *Escherichia*, and *Enterococcus*. Metabolomic analysis revealed a reduction in anti-inflammatory metabolites (Dodecanedioic acid, 5-Phenylvaleric acid, Kynurenic acid) and antioxidant metabolites, coupled with an increase in the pro-inflammatory metabolite Gamma-Glutamyl-L-Putrescine in the HIV-MASLD group. And plasma analysis indicated a decline in 20-carboxy arachidonic acid. Additionally, *Faecalibacterium* and *Anaerobutyricum* correlated positively with anti-inflammatory metabolites, while *Klebsiella*, *Enterobacter*, and *Citrobacter* correlated negatively. Furthermore, *Faecalibacterium* and *Anaerobutyricum* also showed a moderate negative correlation with MDA levels. A random forest model using plasma metabolites showed the optimal discriminative ability for HIV-MASLD (AUC = 0.819 ± 0.0059). Differences in gut microbiota and metabolites in PWHIV may affect MASLD progression by influencing inflammation and oxidative stress. Modulating gut microbiota, such as adding *Faecalibacterium* and *Anaerobutyricum*, could be a treatment strategy for HIV-MASLD. Plasma metabolites have potential as noninvasive markers.

## Introduction

Recent statistics reveal that approximately 39.9 million individuals globally are living with HIV, underscoring AIDS as a persistent and significant challenge in global public health [1]. The implementation of antiretroviral therapy (ART) has led to a gradual decline in AIDS-related mortality rates. Nonetheless, liver diseases continue to be a significant cause of mortality among persons with HIV (PWHIV) [2]. Notably, nonalcoholic fatty liver disease (NAFLD) has surpassed viral hepatitis to become the most prevalent liver disease within the HIV-infected population [3]. In 2020, an international panel of experts proposed renaming NAFLD as MAFLD, which was further updated to metabolic dysfunction-associated steatotic liver disease (MASLD) in 2023 [4].

The global prevalence rate of MASLD in PWHIV is 34%, which exceeds the 30% prevalence observed in the general population [5]. Without timely intervention, MASLD can progress from simple hepatic steatosis to more severe conditions, including nonalcoholic steatohepatitis (NASH), liver cirrhosis, and hepatocellular carcinoma [6]. By 2030, MASLD is projected to become the leading indication for liver transplantation [7]. Additionally, individuals with MASLD are at an increased risk for comorbidities such as cardiovascular disease, type 2 diabetes, and chronic kidney disease [8]. It is important to highlight that the progression from hepatic steatosis to steatohepatitis, liver fibrosis, and other liver-related complications is significantly elevated in PWHIV [9–11].

MASLD is exerting an increasingly significant impact on PWHIV, with a rising global incidence trend [12,13]. Nevertheless, the underlying mechanisms of MASLD in HIV-infected individuals remain inadequately understood, and current pharmacological interventions and treatment strategies are limited. Consequently, further research into these mechanisms is essential for enhancing our understanding of the disease and is crucial for developing effective intervention strategies. Intestinal metabolites serve as a critical medium for the reciprocal regulation between the human host and its microbiota, influencing both health and disease progression. The relationship between intestinal microorganisms and their metabolites, circulating metabolites, and the co-occurrence of HIV infection and MASLD has not been thoroughly investigated. This study aims to characterize the gut microbiota and metabolite profiles in persons with HIV-associated MASLD and to explore the role of microbiota–metabolite interactions in its pathogenesis.

## Materials and methods

### Study design

This two-center, observational, cross-sectional study consecutively screened 186 people with HIV attending outpatient clinics at the First Affiliated Hospital of Zhejiang University School of Medicine and Hangzhou Linping District First People’s Hospital between December 2023 and October 2024. Of these, 88 were excluded during eligibility assessment, including 64 who did not meet the inclusion criteria and 24 who met at least one exclusion criterion. Of the 98 eligible individuals, 10 declined participation, leaving 88 who provided written informed consent and were enrolled. After stool and blood sample collection, 28 participants were excluded due to inadequate stool sample quality, leaving 60 for the final analysis.

Participants were categorized into two groups based on the presence of MASLD: (1) the HIV- MASLD group, comprising individuals with both HIV infection and MASLD (*N* = 36), and (2) the HIV group, consisting of people with HIV alone (*N* = 24).

The inclusion criteria for study participants were as follows: (1) individuals aged 18 years or older with HIV infection; (2) demonstrated good adherence to antiretroviral therapy (ART); (3) a viral load of less than 50 copies/ml, indicating virological suppression; (4) no history of alcohol consumption or daily ethanol intake below 30 g for men and 20 g for women; (5) absence of liver diseases such as viral hepatitis, autoimmune hepatitis, cholestatic liver disease, or Wilson’s disease; and (6) no long-term use of medications associated with steatosis, including glucocorticoids, tamoxifen, amiodarone, sodium valproate, or methotrexate. The exclusion criteria included: (1) Women who are pregnant, breastfeeding, or planning pregnancy; (2) Participants with severe vital organ diseases or impairments; (3) History of gastrointestinal issues, abdominal surgery, or abnormal stool frequency; (4) Recent use of antibiotics or probiotics.

### Data and sample collection

This observational study collected demographic and clinical data from participants, alongside stool and blood samples. Demographic details included age, sex, BMI, waist and hip circumference, HIV diagnosis duration, ART duration, and regimen. Clinical data covered platelet count, liver and kidney function, CD4+T and CD8+T lymphocyte counts, lipid markers, fasting glucose, and history of metabolic conditions. Blood and stool samples were taken under standardized conditions, with participants fasting for 8 hours before blood collection. Blood was centrifuged for plasma storage at −80°C, and fresh stool samples were stored at −80°C within 2 hours.

### Assessment of hepatic steatosis

Eligible study participants underwent fasting transient elastography (FibroScan®) to evaluate liver steatosis. Skilled technicians followed strict protocols, obtaining a minimum of 10 valid measurements per participants. The median controlled attenuation parameter (CAP) in decibels per meter (dB/m) determined the severity: CAP ≥ 238 dB/m indicated fatty liver disease, with 238–259 dB/m as mild, 260–291 dB/m as moderate, and ≥292 dB/m as severe [14,15].

### Gut microbiota assessment

Fecal samples were processed for DNA extraction, PCR amplification, and sequencing library construction. Microbial genomic DNA was extracted using the FastPure Stool DNA Isolation Kit (MJYH, Shanghai, China) as per the manufacturer’s instructions. The DNA’s integrity was checked via 1% agarose gel electrophoresis, and its concentration and purity were measured with a NanoDrop2000 (Thermo Fisher Scientific, USA). Barcoded primers were used for PCR amplification of the full-length 16S rRNA gene. The sequencing library was prepared with the SMRTbell Prep Kit 3.0, involving DNA damage repair, end repair, and adapter ligation, before sequencing.

Samples were differentiated by barcode sequences, filtered by length, and corrected directionally to keep sequences of 1000–1800 bp for bacteria and 300–900 bp for fungi. The DADA2 plugin in Qiime2 was used to denoise these sequences, resulting in ASVs (Amplicon Sequence Variants). Non-compliant sequences were removed. To reduce sequencing depth effects on diversity analyses, sequence counts were normalized to 6000 per sample, maintaining an average coverage of 99.09%. Species taxonomy of ASVs was analyzed with the Naive Bayes classifier in Qiime2 using the Silva 16S rRNA gene database.

Alpha diversity indices (Ace, Chao, Shannon) were calculated using mothur software. Differentially abundant gut microbiota between the two groups were identified using the Wilcoxon rank-sum test, with Benjamini-Hochberg correction for multiple testing. Principal coordinate analysis (PCoA) with the Bray-Curtis distance assessed microbial community structure similarity, while PERMANOVA determined the significance of differences between sample groups. The Wilcoxon signed-rank test compared differentially abundant species, and LEfSe analysis identified significantly different bacterial groups from phylum to species level, using *p* < 0.05 and an LDA score ≥ 3. In this study, LEfSe analysis and Wilcoxon rank-sum test were used for exploratory screening of potential differential microorganisms between the two groups.

### Fecal and serum metabolomic assays

The LC-MS/MS analysis of sample was conducted on a Thermo UHPLC-Exploris240 system equipped with an ACQUITY HSS T3 column (100 mm × 2.1 mm i.d., 1.8 μm; Waters, USA) at Majorbio Bio-Pharm Technology Co. Ltd. (Shanghai, China). The mobile phases consisted of 0.1% formic acid in water: acetonitrile (95:5, v/v) (solventA) and 0.1% formic acid in acetonitrile:isopropanol: water (47.5:47.5, v/v) (solventB). Positive ion mode separation gradient: 0–3 min, mobile phase B was increased from 0% to 20%; 3–4.5 min, mobile phase B was increased from 20% to 35%; 4.5–5 min, mobile phase B was increased from 35% to 100%; 5–6.3 min, mobile phase B was maintained at 100%; 6.3–6.4 min, mobile phase B was decreased from 100% to 0%; 6.4–8 min, mobile phase B was maintained at 0%. Separation gradient in negative ion mode: 0–1.5 min, mobile phase B rises from 0 to 5%; 1.5–2 min, mobile phase B rises from 5% to 10%; 2–4.5 min, mobile phase B rises from 10% to 30%; 4.5–5 min, mobile phase B rises from 30% to 100%; 5–6.3 min, mobile phase B linearly maintains 100%; 6.3–6.4 min, the mobile phase B decreased from 100% to 0%; 6.4–8 min, the mobile phase B was linearly maintained at 0%. The flow rate was 0.40 mL/min and the column temperature was 40°C. QC samples were inserted into the sample cohort to monitor and assess the solidity of the system and the credibility of experimental data.

The ropls package (version 1.6.2) in R was utilized to conduct Principal component analysis (PCA) and orthogonal projections to latent structures discriminant analysis (OPLS-DA) on the preprocessed data matrix. Model stability was evaluated through seven-fold cross-validation. Differential metabolites were screened based on the variable importance in projection (VIP) derived from the OPLS-DA model and statistical difference tests. The Shapiro–Wilk test was applied to evaluate the normality of metabolite data. Student’s t-test was used for normally distributed data, while the Wilcoxon rank-sum test was adopted for non-normal distributed data between the two groups. All *p*-values were corrected using the Benjamini–Hochberg method for multiple testing. Metabolites satisfying VIP > 1 and adjusted *p* < 0.05 were defined as significantly differential metabolites. These differentially expressed metabolites were subsequently annotated for metabolic pathways using the Kyoto Encyclopedia of Genes and Genomes (KEGG) database. Pathway enrichment analysis was conducted using the scipy.stats package in Python, and the biological pathways most pertinent to the experimental treatments were determined through Fisher’s exact test.

### Cytokine and oxidative stress marker analysis in plasma

Cytometric Bead Array (CBA) was used to measure the concentrations of cytokines in the plasma collected from persons enrolled, including IL-4, IL-10, IL-17A and IFN-γ. BD CBA Human Enhanced Sensitivity Master Buffer Kit were used according to manufacturer’s instructions. All antibodies and relative isotype controls were purchased from BD Biosciences (San Diego, CA, USA). The concentrations of superoxide dismutase (SOD) and malondialdehyde (MDA) in the plasma were determined according to the procedures specified in the product manual using enzymatic immunoassay.

### Analysis of clinical variables

Clinical data were analyzed with SPSS version 26. Continuous variables were shown as mean (SD) or median (IQR), and categorical variables as frequency (percentage). Normality was checked with the Shapiro-Wilk test. Depending on normality, continuous variables were tested using the Mann-Whitney U or Student’s t-test, and categorical variables with the chi-square test. Differences with *p*-values < 0.05 were considered statistically significant.

### Correlation analysis among the gut bacteria, metabolites, immune cytokines, and oxidative stress markers

Spearman’s rank correlation analysis was performed to assess associations among gut bacteria, metabolites, immune cytokines, and oxidative stress markers. Spearman’s correlation coefficient was used to evaluate the strength and direction of monotonic relationships between variables. To control for multiple testing, *p* values from correlation analyses were adjusted using the Benjamini–Hochberg false discovery rate method. The results are presented as heatmaps, with significant correlations marked by asterisks (* *p* < 0.05, ** *p* < 0.01, *** *p* < 0.001).

### Random forest classification

A random forest model was developed using the R package random Forest (version 4.7–1.1) to identify potential biomarkers in gut microbiota and plasma metabolites that distinguished the HIV-MASLD group from the HIV group. A total of 60 samples were included in the analysis, with 30 samples in each group. Considering the relatively limited sample size, leave-one-out cross-validation (LOOCV) was adopted for model validation. In each LOOCV iteration, one sample was held out as the testing sample, and the remaining 59 samples were used as the training set. This procedure was repeated 60 times until each sample had been used once as the testing sample. The predicted probabilities of all held-out samples were then aggregated to construct the receiver operating characteristic curve and calculate the area under the curve (AUC).

The number of trees was set to 500, mtry was set to the square root of the number of predictors included in each model, and the terminal node size was set to 1. Because the two groups were balanced, with 30 samples per group, no additional class imbalance correction was applied. If feature selection was performed, it was conducted within each training set during the LOOCV procedure to avoid information leakage from the held-out test sample.

To reduce the influence of stochastic variation inherent to the random forest algorithm, the entire LOOCV procedure was repeated 10 times using different random seeds. For each repeated run, AUC values were calculated based on the predicted probabilities of all held-out samples and summarized as mean ± SD. Single-omic models and the integrated multi-omic model were evaluated using the same LOOCV framework and identical random forest parameter settings. The AUC values were paired according to the same random seed across models and compared using the paired Wilcoxon signed-rank test. A two-sided *p* value < 0.05 was considered statistically significant.

## Results

### Clinical characteristics

Table 1 presents the general characteristics of the participants. Statistical analysis of baseline characteristics indicated that the CAP score for the HIV-MASLD group was 301 dB/m, suggestive of severe hepatic steatosis. Liver function markers, specifically alanine aminotransferase (ALT) and aspartate aminotransferase (AST), were significantly elevated in the HIV-MASLD group compared to the HIV group. Additionally, lipid markers, including triglycerides, total cholesterol, and low-density lipoprotein cholesterol, were higher in the HIV-MASLD group. No statistically significant differences were found between the two groups concerning age, gender, history of metabolic-related diseases, duration of HIV infection, duration of ART and medication regimens.

**Table 1.** Summary of the clinical characteristics.

	HIV (N = 24)	HIV-MASLD (N = 36)	p value
Age (year)	38 (33–47)	37 (32–48)	0.916
Male (%)	23 (95.83)	36 (100.00)	1.000
BMI (kg/m^2^)	23.47 (2.41)	26.02 (3.79)	0.017*
Waist circumference (cm)	89.08 (8.16)	91.97 (9.48)	0.227
Hip circumference (cm)	97.79 (6.53)	98.01 (7.20)	0.904
CAP (dB/m)	208 (182–228)	301 (277–317)	<0.001*
Hypertension (%)	0 (0.00)	4 (11.11)	0.916
Diabetes (%)	0 (0.0)	1 (2.78)	1.000
Cardiovascular disease (%)	0 (0.0)	1 (2.78)	1.000
Years since HIV diagnosis (year)	6 (4)	7 (5)	0.769
Years on ART (year)	6 (4)	7 (5)	0.769
ART regimen			
NRTI+INSTI	18 (75.00)	27 (75.00)	1.000
NRTI+NNRTI	5 (20.83)	9 (25.00)	0.709
NRTI	1 (4.35)	0 (0.00)	0.837
Platelets (×10^9^/L)	213 (41)	225 (55)	0.351
CD3+CD4+ T-cell (cells/µL)	554 (235)	541 (302)	0.849
CD3+CD8+ T-cell (cells/µL)	684 (244)	876 (405)	0.026*
CD4+/CD8+	0.86 (0.34)	0.67 (0.38)	0.004*
TG (mmol/L)	1.39 (0.64)	2.65 (2.34)	0.004*
TC (mmol/L)	3.84 (1.03)	4.63 (1.07)	0.006*
HDL-C (mmol/L)	1.04 (0.24)	0.99 (0.18)	0.391
LDL-C (mmol/L)	2.22 (0.82)	2.77 (0.71)	0.007*
ALT (U/L)	20.50 (16.25–34.00)	38.50 (24.00–60.75)	<0.001*
AST (U/L)	21.00 (17.00–29.25)	27.00 (22.00–39.25)	0.004*
GGT (U/L)	21.50 (17.00–40.05)	35.50 (28.50–69.00)	<0.001*
TBIL (umol/L)	9.75 (7.23–13.60)	11.85 (9.43–15.63)	0.170
Table 1 (cont.)			
	HIV (*N* = 24)	HIV-MASLD (*N* = 36)	*p* value
FBG (mmol/L)	5.36 (5.00–5.90)	5.61 (5.26–6.10)	0.145
UA (umol/L)	368.25 (67.78)	426.07 (106.73)	0.022*
Scr (umol/L)	82.92 (14.28)	82.74 (19.26)	0.969

Data are presented as mean (standard deviation), median (interquartile range) or n (%). BMI body mass index, CAP controlled attenuation parameter, ART antiretroviral therapy, NRTI Nucleoside Reverse Transcriptase Inhibitor, INSTI integrase strand transfer inhibitor, NNRTI non-nucleoside reverse-transcriptase inhibitor, TG triglyceride, TC total cholesterol, HDL-C high-density lipoprotein cholesterol, LDL-C low-density lipoprotein cholesterol, ALT alanine aminotransferase, AST aspartate aminotransferase, GGT gamma-glutamyl transferase, TBIL Total Bilirubin, FBG fasting blood glucose, UA Uric Acid, Scr serum creatinine. * denotes a statistically significant difference.

### Diversity of gut mycobiome in HIV-MASLD participants

To compare gut microbiome characteristics between HIV-MASLD and PWHIV, we collected fecal samples and conducted 16S rRNA gene sequencing. Figure 1(A) shows no significant differences in the Ace (*p* = 0.316), Chao (*p* = 0.298), and Shannon (*p* = 0.850) diversity indices, indicating similar bacterial abundance and diversity in both groups. However, a PCoA based on weighted UniFrac distances (Figure 1(B)) revealed distinct community structures between the two groups (*p* = 0.020). To explore the association between HIV-associated MASLD and gut microbiota, a comparison of ASVs between the HIV and HIV-MASLD groups showed that only 10.76% (324 ASVs) were shared (Figure 1(C)). The HIV-MASLD group had 1,554 unique ASVs (51.63%), while the HIV group had 1,132 unique ASVs (37.61%). Both groups shared dominant phyla such as *Bacillota*, *Pseudomonadota*, *Bacteroidota*, and *Actinomycetota*, and genera like *Blautia*, *Streptococcus*, *Escherichia*, and *Ruminococcus* (Figure 1(D,E)).

**Figure 1.**
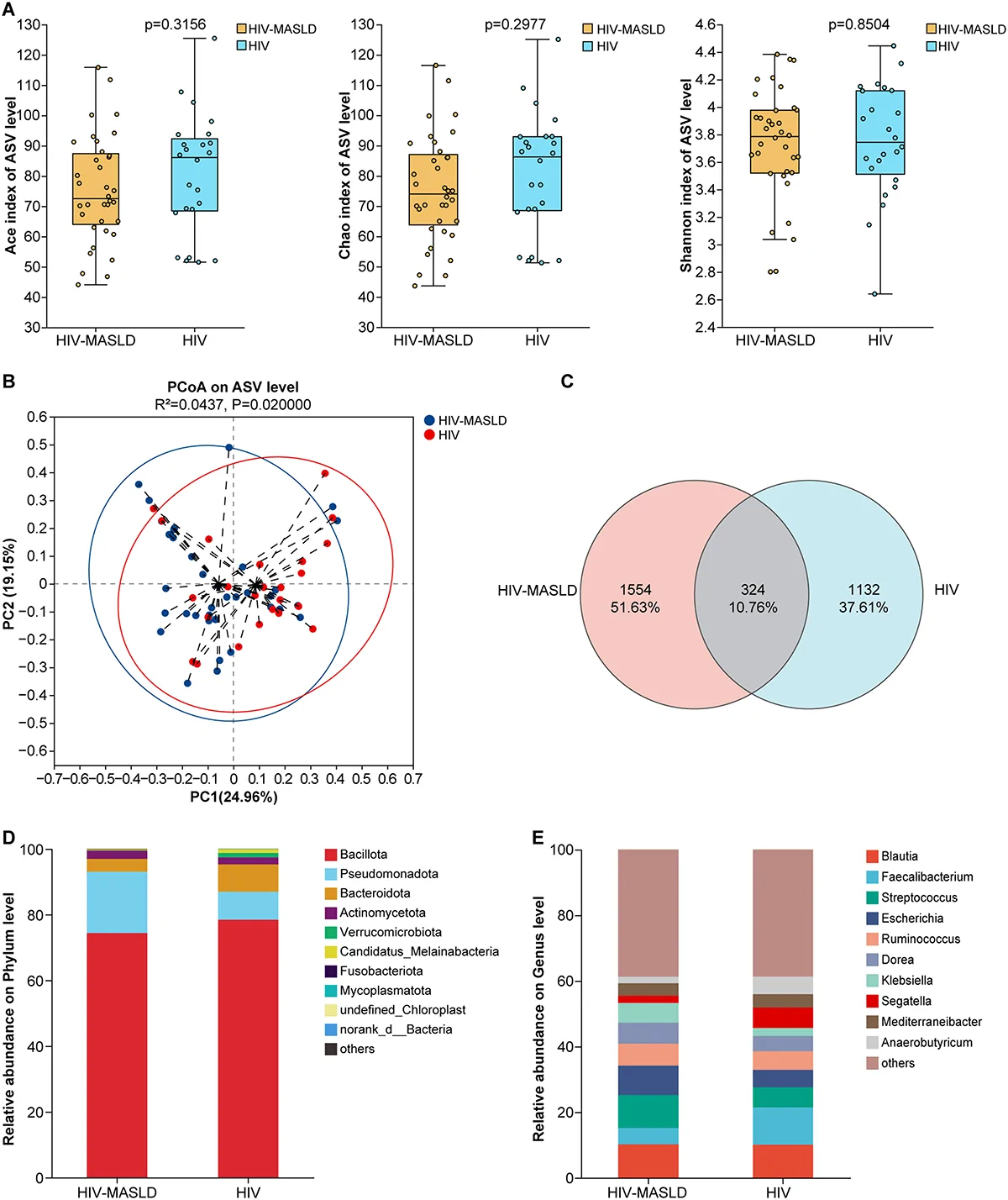
Gut microbiota diversity and composition analysis in HIV-MASLD and HIV groups. (A) alpha diversity comparison using Ace, Chao, and Shannon indices to evaluate species abundance and diversity. (B) beta diversity analysis with PCA based on weighted UniFrac distance and permutation MVA for intergroup differences. (C) venn diagram of shared ASVs. (D, E) Bar charts of the top 10 abundant species at phylum and genus levels for each group.

We compared the top 10 abundant gut microbiota at the phylum, genus, and species levels between the HIV-MASLD and HIV groups. Figure 2(A) indicates that the Proteobacteria phylum was significantly more abundant in the HIV-MASLD group (*p* < 0.050). Among the top 10 genera (Figure 2(B)), Gram-negative bacteria *Klebsiella*, *Dialister*, *Citrobacter*, and *Enterobacter* were notably more abundant in the HIV-MASLD group. The HIV-MASLD group exhibited a significant decrease in beneficial genera *Faecalibacterium* and *Anaerobutyricum*, as well as species *Faecalibacterium prausnitzii* and *Anaerobutyricum hallii* (Figure 2(C)).

**Figure 2.**
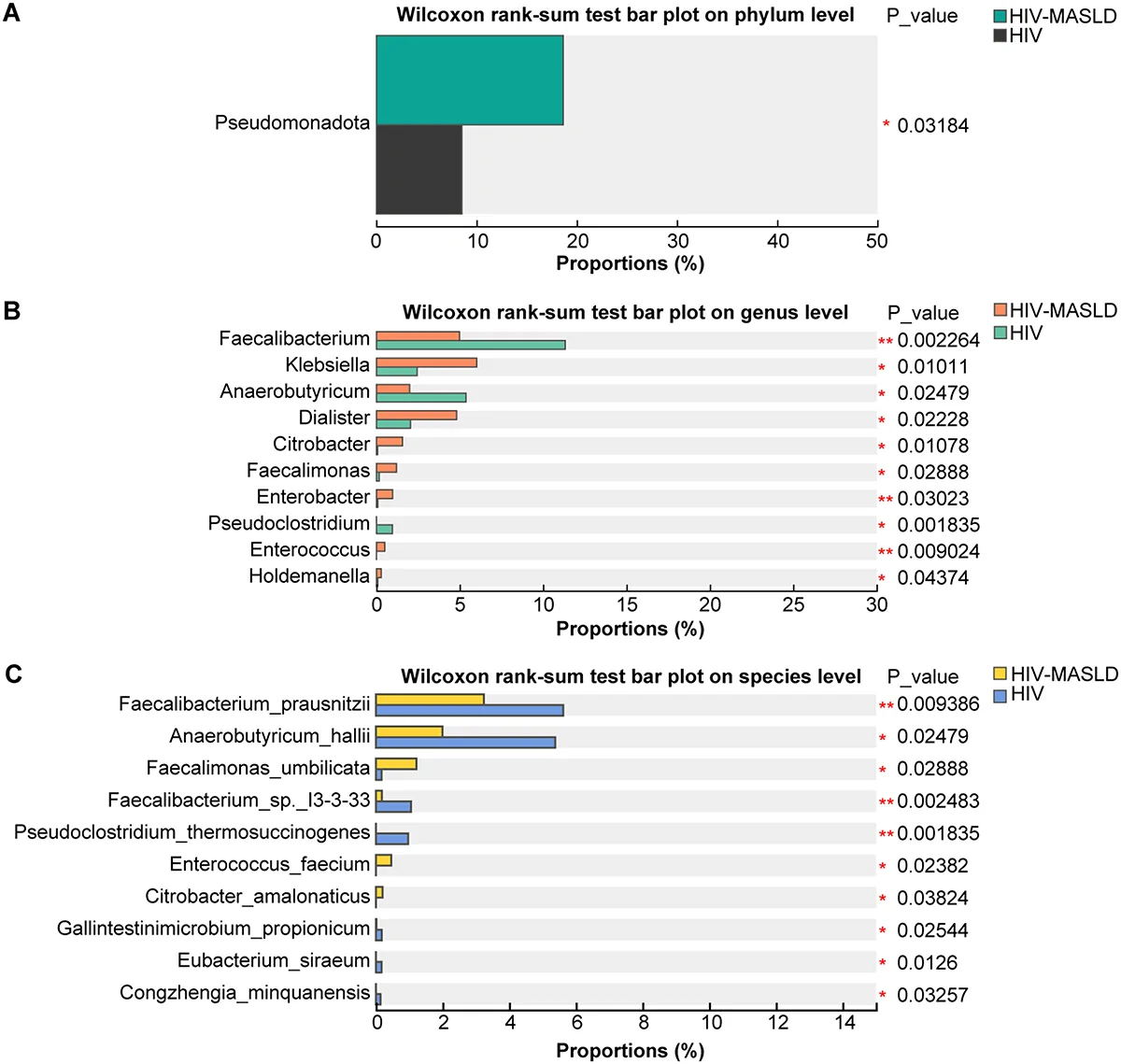
Variations in gut microbiota between HIV-MASLD and HIV groups across three taxonomic levels – genus (A), family (B; displaying the top 10 most abundant genera), and species (C; presenting the top 10 most abundant species).

### Metabolic profiles of gut microbiota and plasma in HIV-MASLD participants

To obtain an unsupervised overview of the metabolomic profiles, PCA was performed. The PCA score plot (Figure 3(A)) showed substantial overlap between the two groups. Subsequently, OPLS-DA (Figure 3(B)) was applied to further explore group-specific metabolic differences between the HIV-MASLD and HIV groups. The OPLS-DA score plot revealed distinct separation between the two groups. Permutation testing confirmed no obvious overfitting (Figure 3(C)), further verifying the robustness of the supervised model. supporting the robustness of the supervised model to some extent. Furthermore, the HIV-MASLD group showed 187 metabolites upregulated and 364 downregulated compared to the HIV group (Figure 3(D)). In comparison to the HIV group, the HIV-MASLD group exhibited a significant downregulation of substances with hepatoprotective properties, including N-Lactoyl-Glycine, 5-Phenylvaleric acid, Dodecanedioic acid, and Kynurenic acid. Conversely, hepatotoxic substances such as Cholesterol and Gamma-Glutamyl-L-Putrescine were significantly upregulated (Figure 3(D)).

**Figure 3.**
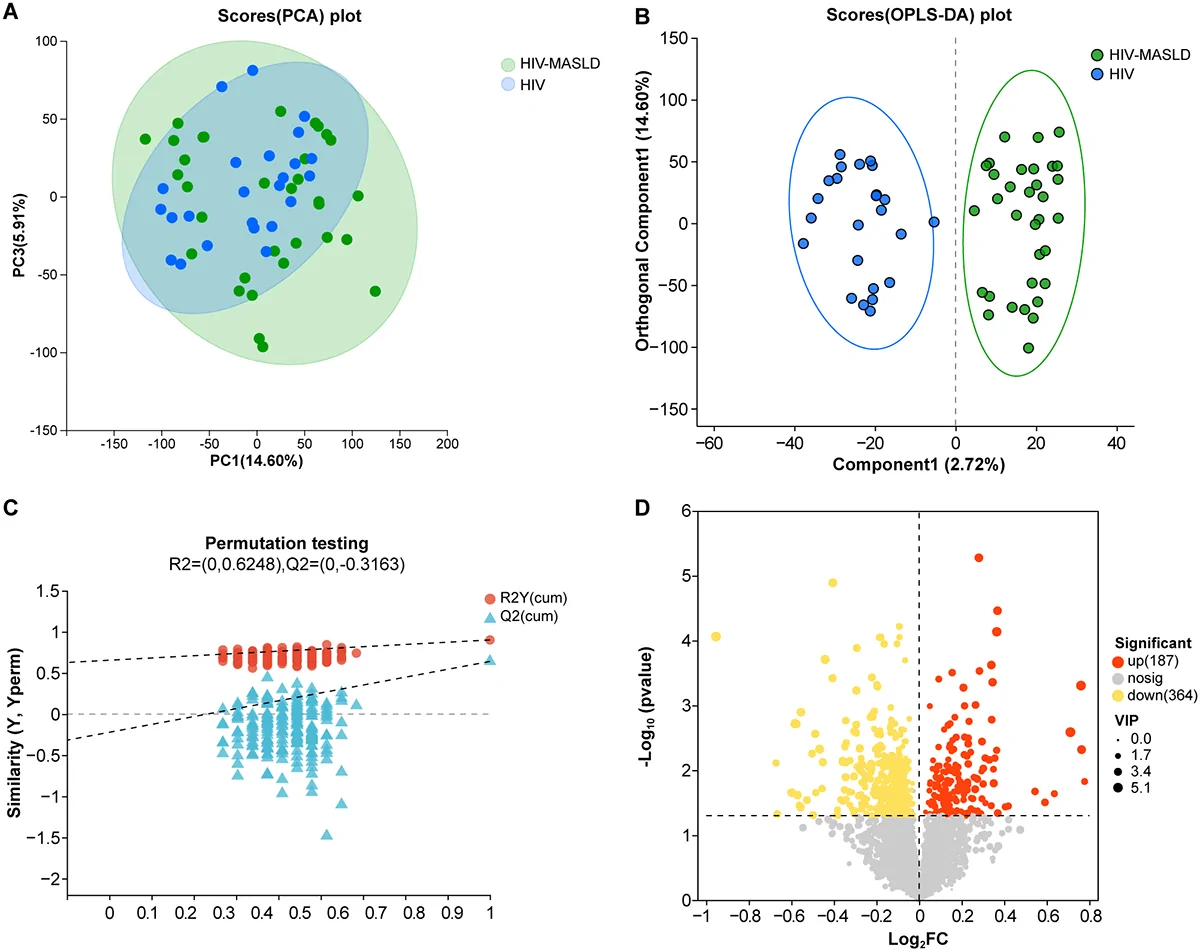
Comparative analysis of fecal metabolites between the HIV-MASLD and HIV groups. (A) PCA score plot. Ellipses represent 95% confidence intervals. (B) OPLS-DA score plot. (C) permutation test of the OPLS-DA model. (D) volcano plot of differential metabolites. Red points indicate upregulated metabolites (*n* = 187); yellow points indicate downregulated metabolites (*n* = 364); point size represents the VIP value.

A PLS-DA model of plasma metabolites (Figure 4(A)) showed distinct differences between the HIV-MASLD and HIV groups. In the combined cation-anion mode, 18 metabolites were upregulated, including Glutamic acid, 2-Ketoglutaric acid, and Succinate Semialdehyde. Furthermore, 105 metabolites were identified as differentially downregulated, such as isolithocholic acid, arachidonic acid, 7-ketolithocholic acid, N-stearoyl tyrosine, 13-L-hydroperoxylinoleic acid, and 20-carboxy arachidonic acid (Figure 4(B)). Further KEGG pathway analysis (Figure 5) identified enriched pathways in both HIV-MASLD and HIV groups, including antioxidant-related pathways like hypoxia-inducible factor-1 (HIF-1), and those related to butyrate metabolism, arginine biosynthesis, and amino acid metabolism. Differential abundance scores (Figure 6) showed significant differences, particularly in antioxidant pathways such as HIF-1 signaling, which were consistently downregulated in the HIV-MASLD group.

**Figure 4.**
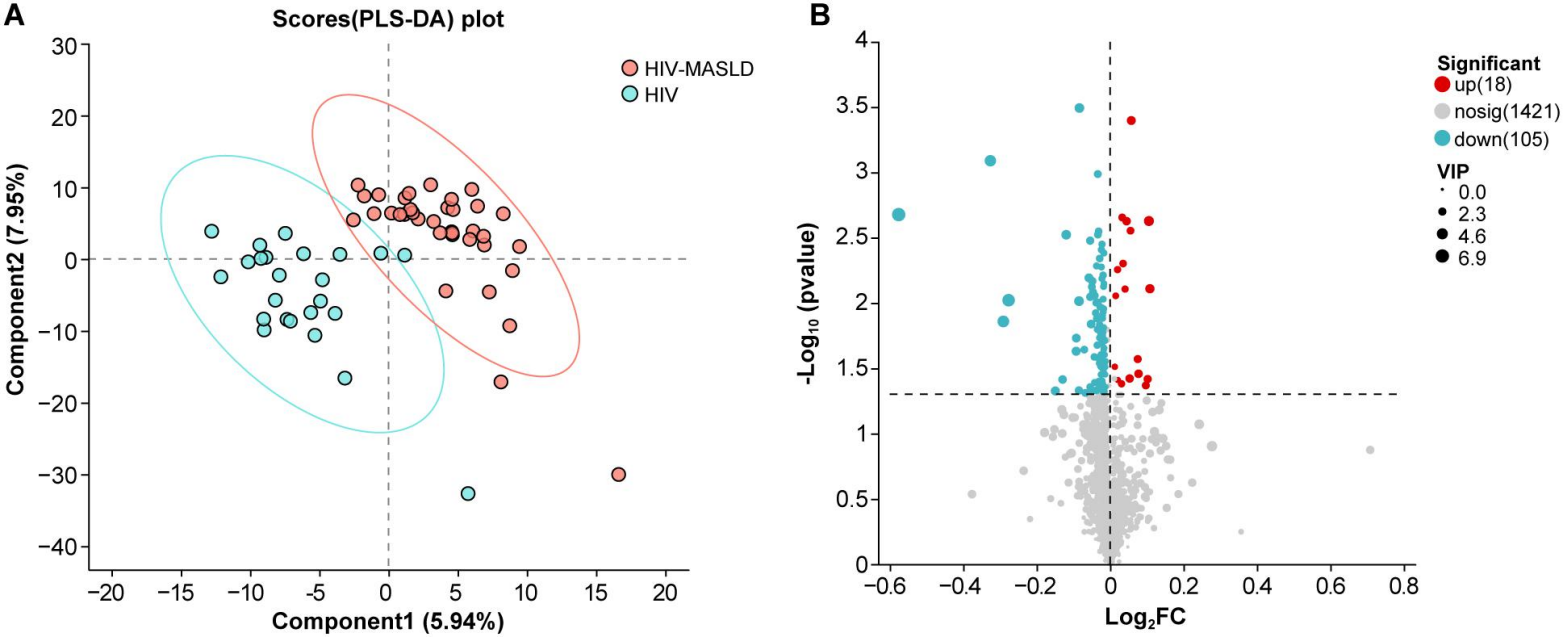
Comparative analysis of plasma metabolite profiles between the HIV-MASLD and HIV groups. (A) OPLS-DA score plot. (B) volcano plot of differential metabolites. Red points indicate upregulated metabolites (*n* = 18); blue points indicate downregulated metabolites (*n* = 105); point size represents the VIP value.

**Figure 5.**
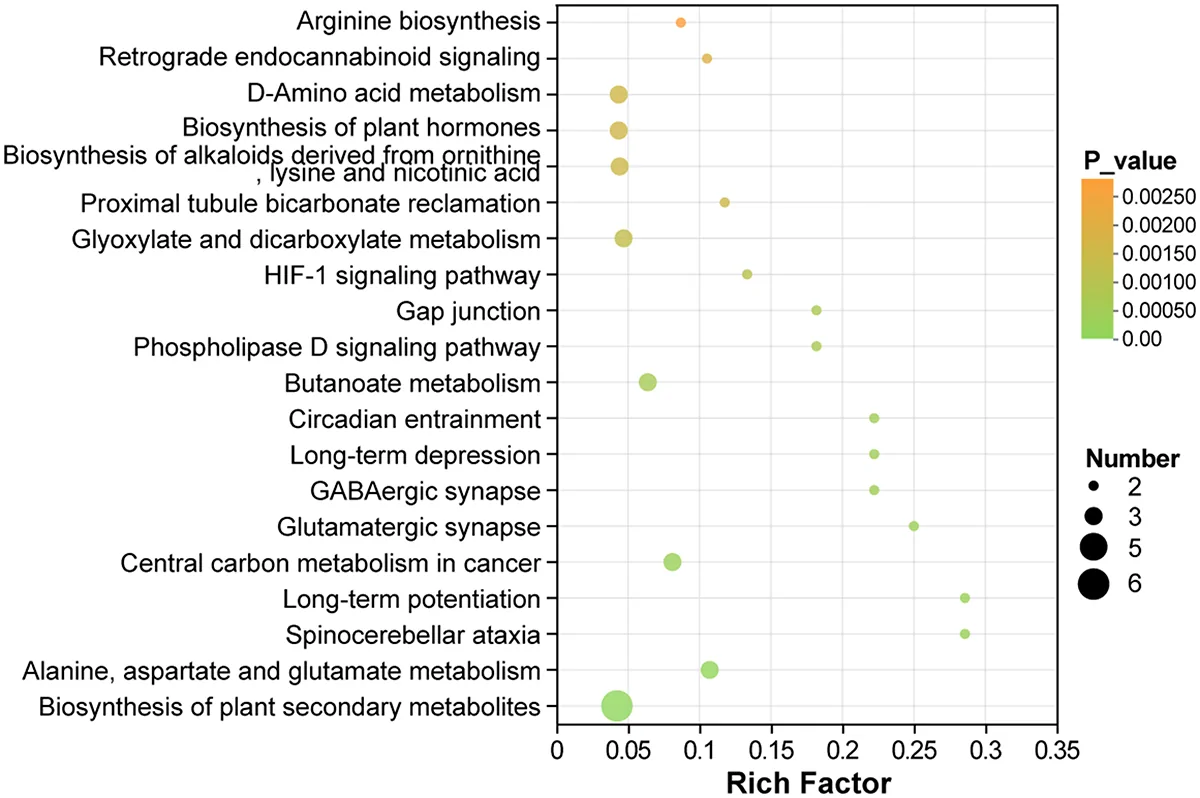
KEGG pathway enrichment of plasma metabolites differing between HIV-MASLD and HIV groups.

**Figure 6.**
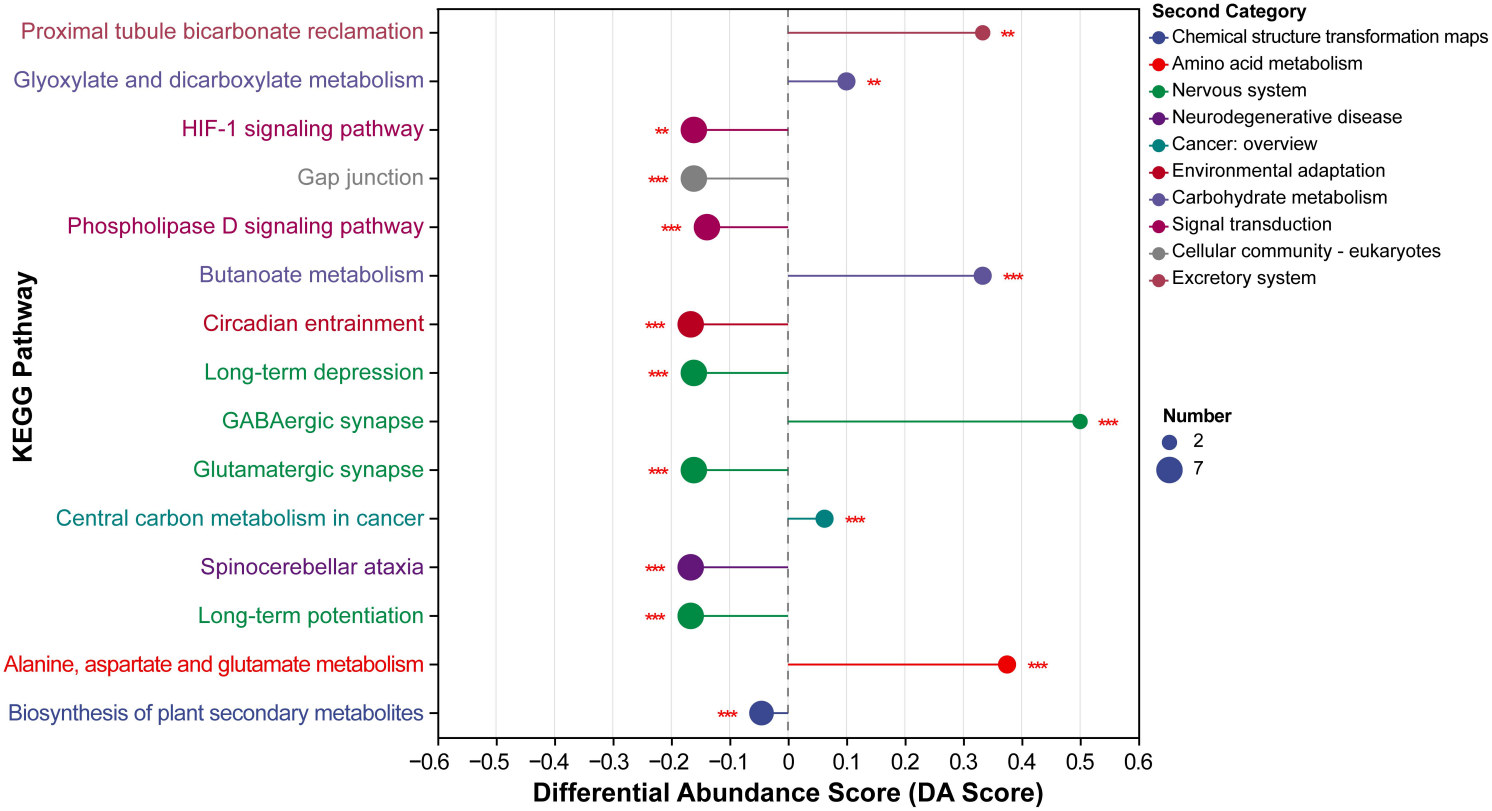
KEGG pathway scores for plasma metabolites differing between HIV-MASLD and HIV groups.

### Associations of gut bacterial and metabolites in HIV-MASLD participants

We conducted Spearman correlation analysis to link bacterial genera with key metabolites that differed between the two groups, examining the interaction between gut microbiota and metabolites. We identified 23 key metabolites across 7 categories (including amino acids, bile acids, and lipids) potentially involved in the pathogenesis of fatty liver disease. Differential microbial communities were linked to gut microbial metabolites (Figure 7 and Supplementary Figure 1). In HIV-MASLD persons, the reduced abundance of *Faecalibacterium* and *Anaerobutyricum* was positively associated with decreased hepatoprotective metabolites like dodecanedioic acid (*p* < 0.001), kynurenic acid (*p* < 0.001), and 5-phenylvaleric acid. In contrast, increased levels of *Klebsiella, Enterobacter*, *Citrobacter*, and *Enterococcus* were negatively associated with these metabolites. Additionally, *Faecalibacterium* and *Anaerobutyricum* were negatively correlated with the hepatotoxic compounds Cholesterol and Gamma-Glutamyl-L-Putrescine, while *Klebsiella*, *Enterobacter*, *Citrobacter*, and *Enterococcus* showed positive correlations with these substances.

**Figure 7.**
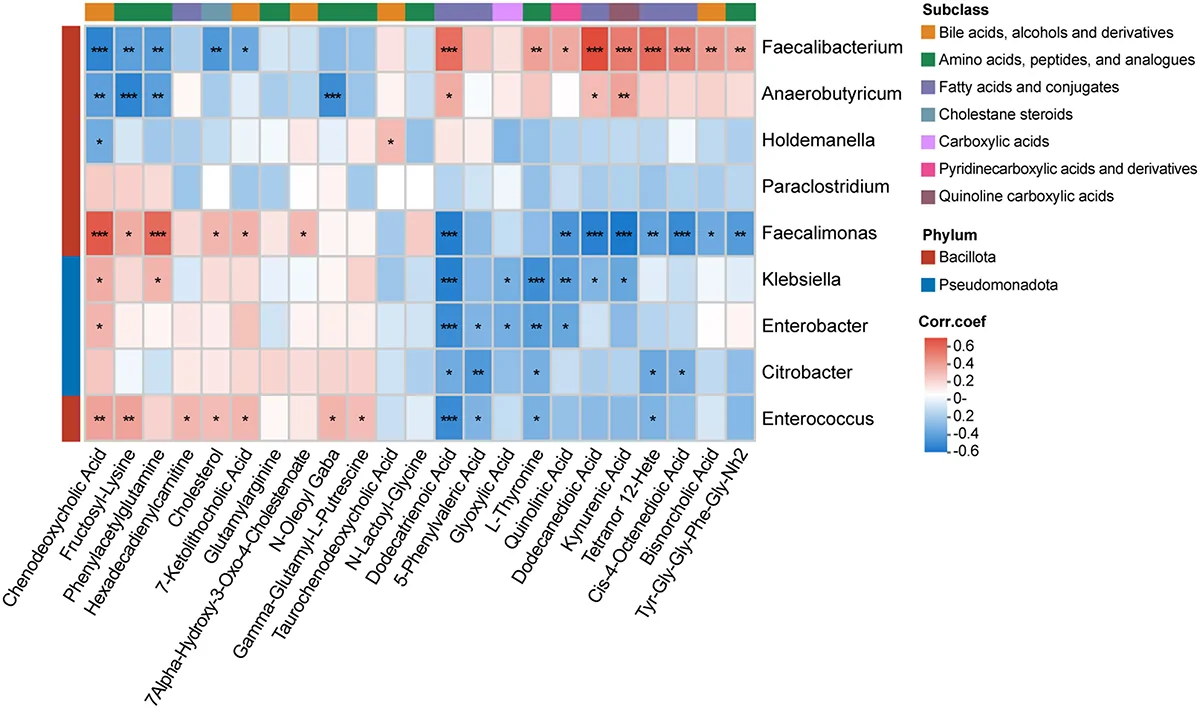
Correlation analysis of key fecal metabolites and bacterial genera in HIV-MASLD. Red shows positive correlations, blue shows negative. Asterisks indicate significant correlations: *p < 0.05, **p < 0.01, ***p < 0.001.

Among the differentially expressed metabolites in plasma, 15 key metabolites spanning 9 categories, including amino acids, bile acids, and unsaturated fatty acids, were identified as potentially implicated in the pathogenesis of fatty liver disease (Figure 8 and Supplementary Figure 2). The results indicated a positive correlation between the beneficial bacterial genera *Anaerobutyricum* and *Faecalibacterium* and hepatoprotective plasma metabolites. In the HIV-MASLD group, there was a noted decrease in the abundance of *Anaerobutyricum* and *Faecalibacterium*, accompanied by a significant downregulation of the hepatoprotective metabolite 20-Carboxy Arachidonic acid. Conversely, potentially pathogenic bacterial genera exhibited negative correlations with hepatoprotective plasma metabolites. Pathogenic bacteria like *Klebsiella*, *Citrobacter*, and *Enterobacter* increased, while N-Stearoyl Tyrosine, Gamma-Tocopherol, and 20-Carboxy Arachidonic acid were downregulated.

**Figure 8.**
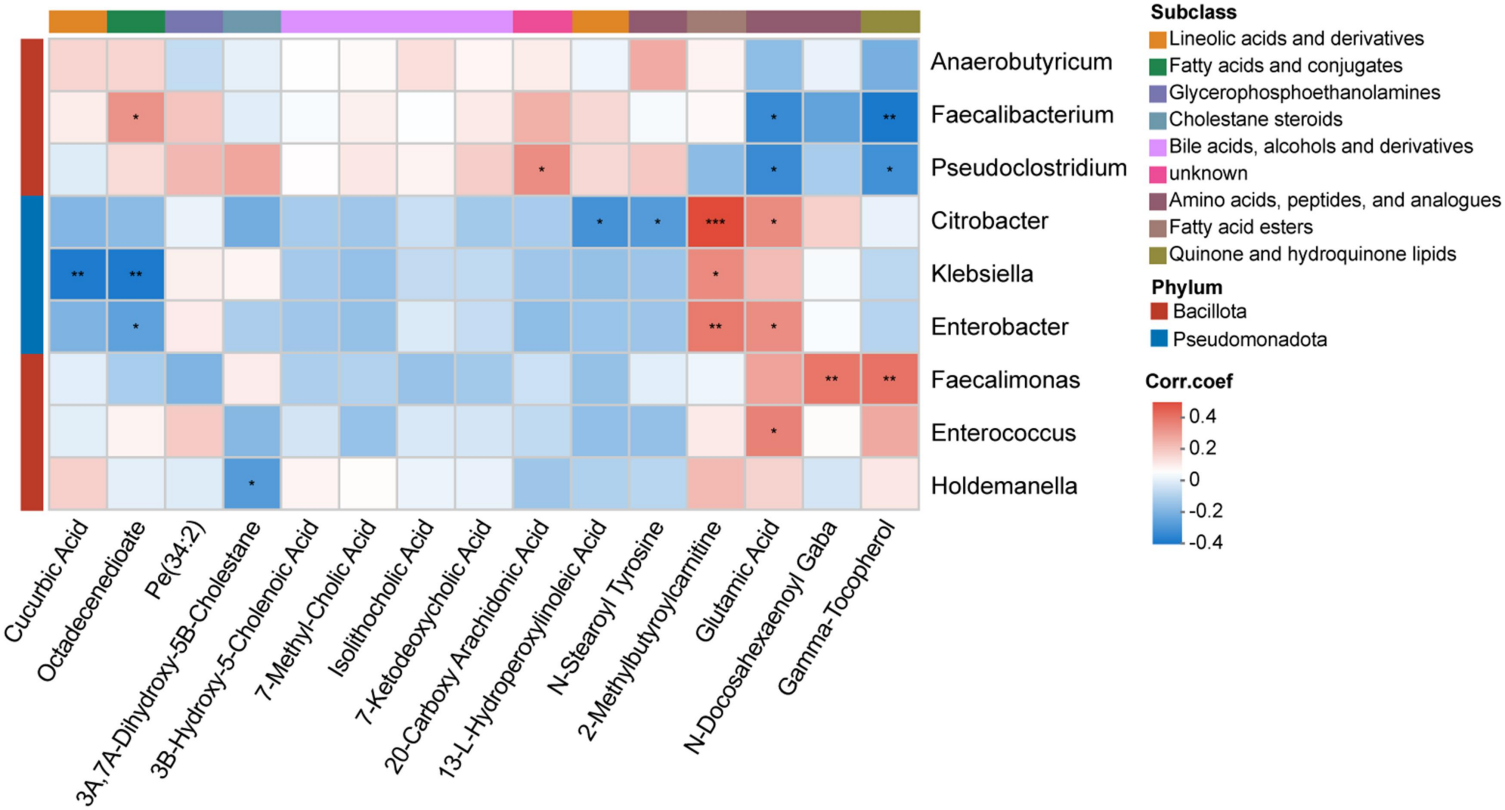
Correlation analysis of key plasma metabolites and bacterial genera in HIV-MASLD. Red shows positive correlations, blue shows negative. Asterisks indicate significant correlations: *p < 0.05, **p < 0.01, ***p < 0.001.

### Correlation analysis of gut microbiota, inflammatory factors, and oxidative stress markers

We conducted an evaluation of the correlations between the top 20 phyla and genera across all participants and the levels of inflammatory cytokines, in addition to the level of SOD and MDA. *Pseudomonadota* abundance showed a significant positive correlation with IFN-γ (*r* = 0.3, *p* < 0.05) at the phylum level in Figure 9(A). And *Fusobacteriota* showed a positive correlation with the level of IL-4, IL-10, IL-17A, and IFN-γ, with a significant correlation to the level of IL-17A (*r* = 0.3, *p* < 0.05) in Figure 9(A). While *Bacteroidota* exhibited a significant negative correlation with the level of SOD (*r* = −0.3, *p* < 0.05). Further analysis revealed correlations between the top 20 bacterial genera and selected indicators. *Streptococcus* positively correlated with the level of IL-4, IL-10, IL-17A, MDA, and SOD, notably with MDA (*r* = 0.3, *p* < 0.05) (Figure 9(B)). *Segatella* negatively correlated with the level of SOD (*r* = −0.3, *p* < 0.05), and *Dialister* negatively correlated with the level of MDA (*r* = −0.4, *p* < 0.001) (Figure 9(B)). The beneficial genera *Faecalibacterium* and *Anaerobutyricum* exhibited a moderate negative correlation with the level of MDA (*r* = −0.2) in Figure 9(B) and Supplementary Figure 3.

**Figure 9.**
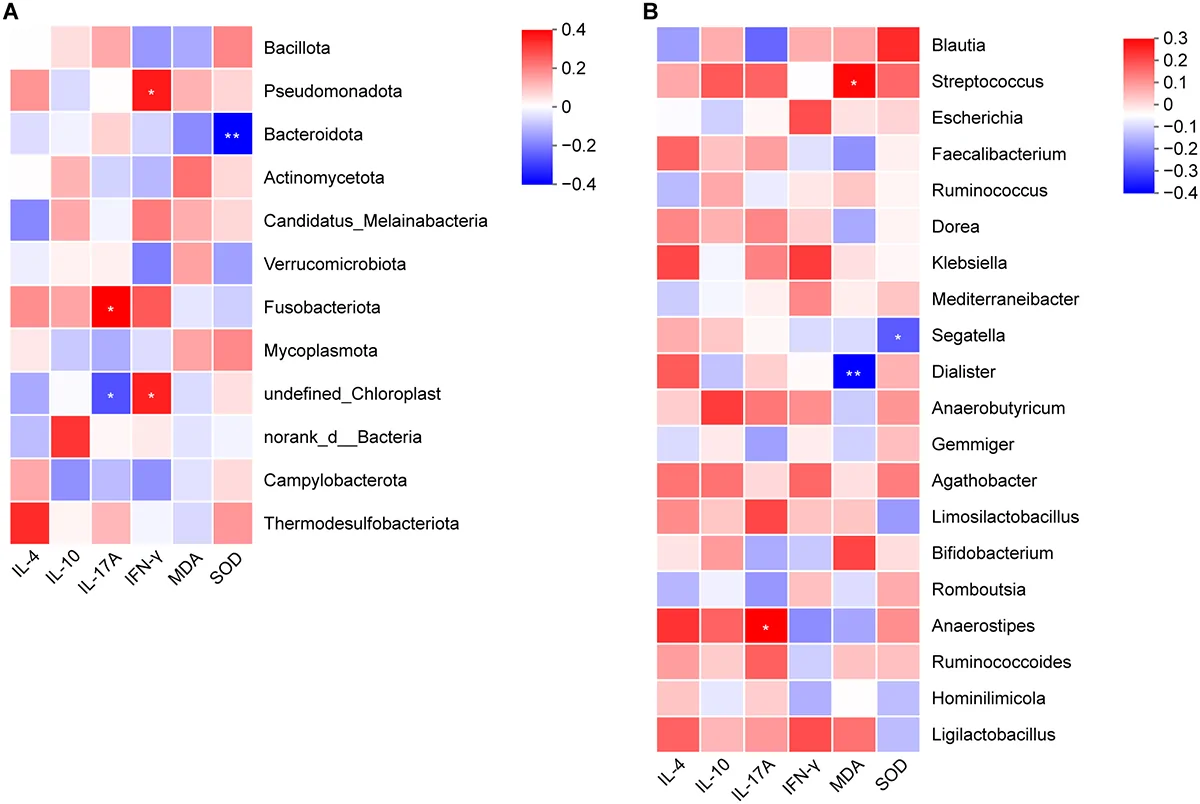
The relationship between intestinal microbiota and specific indicators was examined. The Spearman correlations between the selected indicators and the top 20 abundant phyla (heatmap A) and genera (heatmap B) are depicted in the heatmaps. sod: superoxide dismutases; MDA: Malondialdehyde. *P < 0.05; **P < 0.001; ***P < 0.0001.

### HIV-MASLD diagnosis based on multiomics data

To identify potential diagnostic biomarkers, we developed a random forest classifier utilizing genus-level differences in gut microbiota and plasma metabolites between the HIV-MASLD group and the HIV group. The classification performance of this model was assessed using ROC curves, as shown in Figure 10(A). The plasma metabolomic model showed the best discriminative performance, with an AUC of 0.819 ± 0.0059. The gut microbiota model showed relatively limited classification ability, with an AUC of 0.643 ± 0.084, while the integrated multi-omic model achieved an AUC of 0.783 ± 0.0059. Further comparison of AUC values showed that the plasma metabolomic model performed significantly better than both the gut microbiota model and the integrated multi-omic model (*p* < 0.05) in Figure 10(B). These results suggest that plasma metabolites have strong potential for distinguishing participants with HIV-MASLD from those with HIV alone, whereas gut microbiota features alone showed relatively modest discriminative ability.

**Figure 10.**
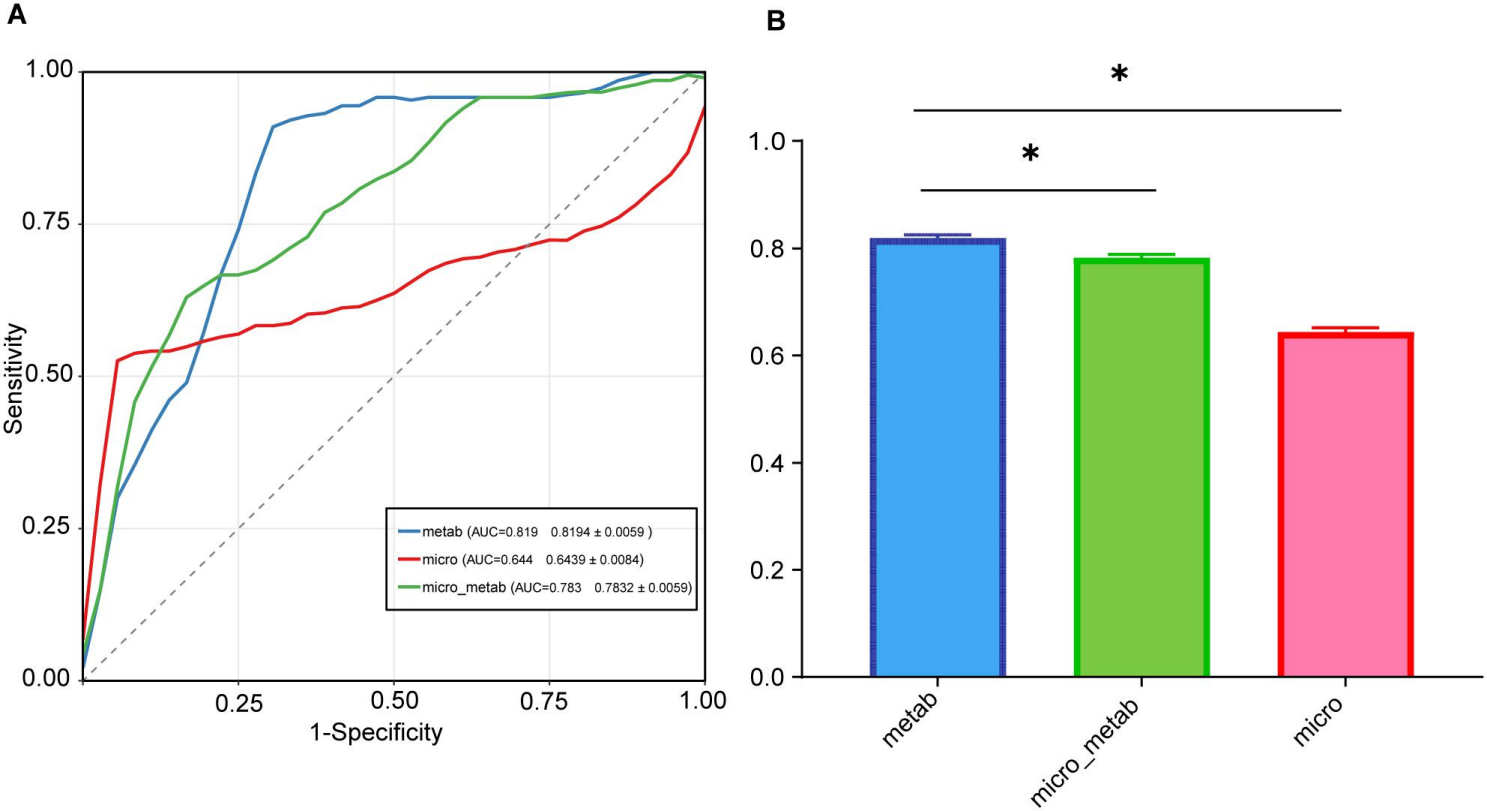
Evaluation of the classification performance of random forest models for HIV-MASLD. (A) ROC curve analysis of the microbiome and plasma metabolites classification model. (B) comparison of AUC values among different omic models. Differences between models were assessed using the paired Wilcoxon signed-rank test. *p < 0.05.

## Discussion

MASLD is often underestimated and poses risks to liver health and other diseases [8]. HIV-infected individuals have a higher prevalence and risk of severe MASLD compared to the general population [7,9]. However, there are limited targeted treatments for HIV-related MASLD, which has a complex, multifactorial pathogenesis. Numerous studies link gut microbiota to fatty liver disease, but research on this in PWHIV with MASLD is limited [16]. Metabolomics, which detects metabolism-related molecules, can help uncover disease mechanisms. To better understand HIV-MASLD pathogenesis and find biomarkers, we combined gut microbiome analysis with LC-MS non-targeted metabolomics. This approach aims to improve early diagnosis and develop personalized interventions for HIV-MASLD.

In our study, the gut microbiota alpha diversity in the HIV-MASLD group was similar to the HIV group, aligning with previous findings on MASLD in PWHIV [17]. However, a Spanish study found that HIV-positive individuals without MASLD had significantly lower gut microbial alpha diversity than those with MASLD alone or PWHIV with MASLD [17]. This difference might be due to variations in Mediterranean diets, geographical environments, or ART regimens. This study found no reduction in alpha diversity of gut microbiota in PWHIV, but significant differences in microbiota composition and structure were observed. Differences in specific bacterial genera may indicate disease status. Sequencing showed a decrease in beneficial *Faecalibacterium* and *Anaerobutyricu* and an increase in potentially harmful *Klebsiella*, *Enterobacter*, *Citrobacter*, and *Enterococcus*. *Faecalibacterium*‘s anti-inflammatory benefits are well-documented, and its reduced presence may lead to intestinal inflammation [18]. Chronic gut inflammation can result in excessive reactive oxygen species production and insufficient antioxidant response, causing ongoing oxidative stress [19,20]. Studies indicate that MASLD persons have reduced levels of *Faecalibacterium* in their gut, unlike PWHIV who show no significant difference compared to healthy individuals [17,21]. *Anaerobutyricum*, a butyrate-producing genus in the Firmicutes phylum, is vital for gut health. Butyrate provides energy for intestinal cells, maintains the anaerobic environment by regulating HIF, strengthens the intestinal barrier by modulating Claudin-1 and synaptopeptin, reduces pro-inflammatory cytokines such as interleukin-6 (IL-6) and IL-12, and alleviates oxidative stress [22–24]. Dysbiosis of gut microbiota, marked by reduced *Faecalibacterium* and *Anaerobutyricum*, can weaken the intestinal barrier and allow bacterial translocation, leading to chronic liver inflammation, oxidative stress, and lipid metabolism disorders, contributing to MASLD [25]. *Anaerobutyricum* levels are similar in both HIV-negative and HIV-positive MASLD persons [17,21]. Within the Proteobacteria phylum, Gram-negative bacteria like *Klebsiella*, *Enterobacter*, and *Citrobacter* activate signaling pathways, such as Toll-like receptor 4, by releasing lipopolysaccharides, which trigger inflammatory cytokines and local gut inflammation [26]. These processes can also cause liver and systemic inflammation via the gut-liver axis, worsening hepatic steatosis. In conclusion, we found distinct compositional changes in the gut microbiota of PWHIV with MASLD, which may worsen lipid metabolism disorders by causing intestinal inflammation and oxidative stress, thus contributing to HIV-associated MASLD [25,27]. While further validation is needed, our multi-omics study offers insights for future research on the role of gut microbes and metabolites in this condition.

While changes in the microbiome are linked to MASLD development, there is limited data on the gut microbiome in PWHIV with MASLD. Most studies have focused on gut microbiota characterization without examining its impact on intestinal and systemic metabolism [28]. Since fecal and plasma metabolites are key mediators of microbiota’s effects on the host, understanding metabolic pathways is essential. Thus, we performed non-targeted metabolomics on fecal and plasma samples from our study participants to investigate metabolite differences and their functional significance. PCA showed significant overlap between the groups, indicating similar metabolic baselines, while OPLS-DA clearly separated them. This suggests that the groups share consistent global metabolic profiles, with differences primarily in specific metabolites. The results of fecal metabolomics analysis identified several metabolites linked to inflammation and oxidative stress, including N-Lactoyl-Glycine, 5-Phenylvaleric acid, Dodecanedioic acid, Kynurenic acid, and Gamma-Glutamyl-L-Putrescine. These metabolic alterations indicate that dysbiosis of the gut microbiota is intricately associated with host inflammatory responses and oxidative stress levels, potentially contributing to the pathogenesis of hepatic steatosis. In a mouse model of diet-induced hepatic steatosis, levels of glycine – a precursor of the endogenous antioxidant reduced glutathione – and its derivative N-lactoyl-glycine were significantly decreased, implicating oxidative stress in the development of hepatic steatosis [29]. 5-Phenylvaleric acid, a medium-chain fatty acid, offers neuroprotection in neurodegenerative diseases by modulating endoplasmic reticulum stress and may also protect the liver [30,31]. Dodecanedioic acid reverses liver inflammation and steatosis in mice [32]. Kynurenic acid, a tryptophan metabolite, has anti-inflammatory properties and aids in regulating liver lipid metabolism and inflammation [33–37]. Elevated Gamma-Glutamyl-L-Putrescine levels are linked to intestinal inflammation in mice with ulcerative colitis, while probiotics reduce these levels and alleviate inflammation in kittens [38]. Previous studies focused on animal models, but our research found changes in key fecal metabolites in PWHIV. We discovered that *Faecalibacterium* and *Anaerobutyricum*, which are reduced in HIV-MASLD participants, positively correlate with beneficial metabolites like dodecanedioic acid and kynurenic acid. In contrast, *Klebsiella*, *Enterobacter*, *Citrobacter*, and *Enterococcus* are negatively correlated with these beneficial metabolites. Additionally, *Faecalibacterium* and *Anaerobutyricum* demonstrated negative correlations with the hepatotoxic compounds cholesterol and gamma-glutamyl-L-putrescine, whereas *Klebsiella*, *Enterobacter*, *Citrobacter*, and *Enterococcus* showed positive correlations with these substances. Recent research on gut microbiota and metabolomics in MASLD participants has mainly focused on the link between gut bacteria and lipid metabolites [39]. Our study indicates that in HIV-MASLD participants, specific bacterial taxa, including *Anaerobutyricum* and members of the *Proteobacteria phylum*, might contribute to hepatic steatosis by affecting inflammation and oxidative stress-related metabolites. Compared with HIV-uninfected individuals, persistent immune activation and intestinal barrier dysfunction in PWHIV may enhance microbial translocation and amplify microbiota-mediated inflammatory and oxidative stress responses. In addition, HIV- and ART-associated metabolic abnormalities may further interact with gut dysbiosis. Therefore, these HIV-specific factors may contribute to a distinct gut microbiota–SLD interplay in PWHIV [3].

We further investigated the effect of gut microbiota on host metabolism using plasma metabolomics in both HIV-MASLD and HIV groups. KEGG pathway analysis of differential metabolites revealed enriched pathways, including those related to short-chain fatty acids, amino acids, and antioxidant signaling such as the HIF-1 signaling pathway, which demonstrated a trend of downregulated expression in the HIV-MASLD group. HIF-1, a hypoxia-inducible factor, is involved in various signaling pathways, enhancing lipid metabolism, regulating mitochondria, and reducing oxidative stress to protect cells [40]. In PWHIV with MASLD, HIF-1 may play a protective role, with its downregulation worsening oxidative stress, fat accumulation, and liver inflammation. Our analysis showed that lower levels of *Anaerobutyricum* and *Faecalibacterium* in HIV-MASLD participants were linked to reduced 20-Carboxy Arachidonic acid. The increased abundance of *Klebsiella*, *Citrobacter*, and *Enterobacter* exhibited a negative correlation with the downregulation of gamma-tocopherol and 20-carboxy arachidonic acid. Notably, 20-carboxy arachidonic acid functions as a dual activator of peroxisome proliferator-activated receptors alpha and gamma, thereby contributing to the reduction of blood lipid levels [41]. Gamma-tocopherol, an isomer of vitamin E, is recognized for its significant antioxidant properties and exerts protective effects through its anti-inflammatory and antioxidant mechanisms. Empirical evidence suggests that gamma-tocopherol mitigates hepatic lipid accumulation, enhances liver function markers, and plays a preventive and therapeutic role in metabolic liver diseases like MASLD [42,43]. Our research demonstrated that the alterations in these protective plasma-circulating metabolites occur concurrently with a decrease in beneficial bacteria and an increase in potentially pathogenic bacteria within the gut microbiota. In PWHIV with MASLD, the majority of the altered metabolites identified in both fecal and plasma metabolomic profiles were linked to inflammation and oxidative stress. This phenomenon can be attributed to the impact of HIV infection on the immune system, resulting in immunosuppression, exacerbated inflammatory pathways, and heightened oxidative stress damage. Consequently, this contributes to the elevated prevalence of fatty liver disease and the increased risk of disease progression observed in PWHIV. These findings offer new understanding of how gut microbiota and metabolism contribute to MASLD in PWHIV, linked to inflammation and oxidative stress. Future research could culture key microbes for in vitro testing to confirm their impact on inflammation and oxidative stress. Additionally, targeted metabolomics could support these findings, laying the groundwork for microbial interventions.

We analyzed inflammatory cytokines (IL-4, IL-10, IL-17A, IFN-γ) and oxidative stress markers (SOD, MDA), and investigated their correlations with the top 20 abundant phyla and genera. IFN-γ, crucial for T helper type 1 immune responses and macrophage activation, showed a positive correlation with *Pseudomonadota* and *Streptococcus*, indicating these microbes might influence immune cell activation [44]. SOD is a key antioxidant enzyme that removes superoxide anion free radicals [45], and its positive link with Bacteroidota suggests the bacteria may aid antioxidation. MDA, a major lipid peroxidation byproduct, serves as a classic oxidative damage marker [46]. Our findings show *Faecalibacterium* and *Anaerobutyricum* moderately negatively correlate with MDA, suggesting that boosting these beneficial bacteria could lower oxidative stress, supporting previous research. We compared six target detection indicators between two groups and found no statistical significance, suggesting individual variability in these biomarkers. Average levels may be influenced by factors like the constant immune activation and regulation in HIV-infected individuals, whose complex compensatory mechanisms can impact marker levels.

FibroScan is a rapid and accurate noninvasive tool for assessing hepatic steatosis and fibrosis. Our study found that gut microbiota and plasma metabolites may serve as complementary molecular biomarkers for PWHIV with MASLD, based on omics data. Recent research has focused on noninvasive biomarkers to differentiate NAFLD, NASH, and liver fibrosis in general population [47,48]. These molecular biomarkers may provide additional information for disease characterization and risk stratification alongside established noninvasive tools.

This study has several limitations. First, convenience sampling from two centers, a small sample size, and the cross‑sectional design may introduce selection bias, reduce generalizability, and preclude causal inferences. Larger‑scale, multicenter prospective cohorts are needed to mitigate bias and establish causality. Second, HIV‑negative MASLD and healthy control groups were absent in our cohort. We therefore performed comparative analyses based on published gut‑microbiota data from these populations. Multi‑group controls should be included in future investigations to profile gut microbiota and metabolites across distinct populations. Third, although LEfSe and the Wilcoxon rank‑sum test are suitable for preliminary screening of candidate microbial biomarkers, they cannot fully account for microbiome‑specific biases including compositional effects, high sparsity, and uneven sequencing depth. Given that analytical approaches may yield divergent outputs, the differential taxa reported here should be regarded as preliminary findings, which require further validation using larger cohorts and more robust compositional data analysis workflows.

## Conclusion

Specific gut bacteria might affect inflammation and oxidative stress by regulating metabolic products. Modulating gut microbiota, such as adding beneficial bacteria like *Faecalibacterium* and *Anaerobutyricum*, could be a potential treatment for HIV-MASLD. The model based on gut microbiota and plasma metabolites showed strong predictive ability for diagnosing HIV-MASLD, offering insights for developing noninvasive diagnostic biomarkers.

## Supplementary Material

ID255997685 Supplementary Material.doc

## Data Availability

The data that support the findings of this study are available in figshare at https://doi.org/10.6084/m9.figshare.32400012 [47].
