# Supplementary material for: Gut microbiota and metabolic characteristics in PWHIV with metabolic dysfunction-associated steatotic liver disease (MASLD)
Source: Virulence. 2026 Oct 5;18(1):2739091. doi: 10.1080/21505594.2026.2739091 (PMC13644625; doi:10.1080/21505594.2026.2739091)
Supplement: Supplementary file 1 [file KVIR_A_2739091_SM7113.doc]

Supplementary Figure 1: Correlation analysis of key fecal metabolites metabolites and bacterial genera in HIV group. Red shows positive correlations, blue shows negative. Asterisks indicate significant correlations: * p<0.05, ** p<0.01, *** p<0.001.

Supplementary Figure 2: Correlation analysis of key plasma metabolites and bacterial genera in HIV group. Red shows positive correlations, blue shows negative. Asterisks indicate significant correlations: * p<0.05, ** p<0.01, *** p<0.001.

Supplementary Figure 3: The Spearman correlations between the selected indicators and the top 20 abundant genera are depicted in the heatmaps. SOD: Superoxide Dismutases; MDA: Malondialdehyde. ∗P<0.05; ∗∗P<0.001; ∗∗∗P<0.0001.
